# Phylogenetic relationships of Malaysia’s long-tailed macaques, *Macaca fascicularis*, based on cytochrome *b* sequences

**DOI:** 10.3897/zookeys.407.6982

**Published:** 2014-05-08

**Authors:** Muhammad Abu Bakar Abdul-Latiff, Farhani Ruslin, Vun Vui Fui, Mohd-Hashim Abu, Jeffrine Japning Rovie-Ryan, Pazil Abdul-Patah, Maklarin Lakim, Christian Roos, Salmah Yaakop, Badrul Munir Md-Zain

**Affiliations:** 1 School of Environmental and Natural Resource Sciences, Faculty of Science and Technology, Universiti Kebangsaan Malaysia, 43600, Bangi, Selangor, Malaysia; 2 UCSI University, 1, Jalan Menara Gading, UCSI Heights, 56000, Cheras, Kuala Lumpur, Malaysia; 3 Department of Wildlife and National Parks, Km 10, Jalan Cheras, 50664, Kuala Lumpur, Malaysia; 4 Sabah Parks, Research and Education Division, PO Box 10626, 88806, Kota Kinabalu, Sabah, Malaysia; 5 Gene Bank of Primates, German Primate Center, Leibniz Institute for Primate Research, 37077 Göttingen, Germany

**Keywords:** Long-tailed macaque, *Macaca fascicularis*, Cytochrome *b*, phylogenetic relationships

## Abstract

Phylogenetic relationships among Malaysia’s long-tailed macaques have yet to be established, despite abundant genetic studies of the species worldwide. The aims of this study are to examine the phylogenetic relationships of *Macaca fascicularis* in Malaysia and to test its classification as a morphological subspecies. A total of 25 genetic samples of *M. fascicularis* yielding 383 bp of Cytochrome *b* (Cyt *b*) sequences were used in phylogenetic analysis along with one sample each of *M. nemestrina* and *M. arctoides* used as outgroups. Sequence character analysis reveals that Cyt *b* locus is a highly conserved region with only 23% parsimony informative character detected among ingroups. Further analysis indicates a clear separation between populations originating from different regions; the Malay Peninsula versus Borneo Insular, the East Coast versus West Coast of the Malay Peninsula, and the island versus mainland Malay Peninsula populations. Phylogenetic trees (NJ, MP and Bayesian) portray a consistent clustering paradigm as Borneo’s population was distinguished from Peninsula’s population (99% and 100% bootstrap value in NJ and MP respectively and 1.00 posterior probability in Bayesian trees). The East coast population was separated from other Peninsula populations (64% in NJ, 66% in MP and 0.53 posterior probability in Bayesian). West coast populations were divided into 2 clades: the North-South (47%/54% in NJ, 26/26% in MP and 1.00/0.80 posterior probability in Bayesian) and Island-Mainland (93% in NJ, 90% in MP and 1.00 posterior probability in Bayesian). The results confirm the previous morphological assignment of 2 subspecies, *M. f. fascicularis* and *M. f. argentimembris*, in the Malay Peninsula. These populations should be treated as separate genetic entities in order to conserve the genetic diversity of Malaysia’s *M. fascicularis*. These findings are crucial in aiding the conservation management and translocation process of *M. fascicularis* populations in Malaysia.

## Introduction

**Figure 1.**
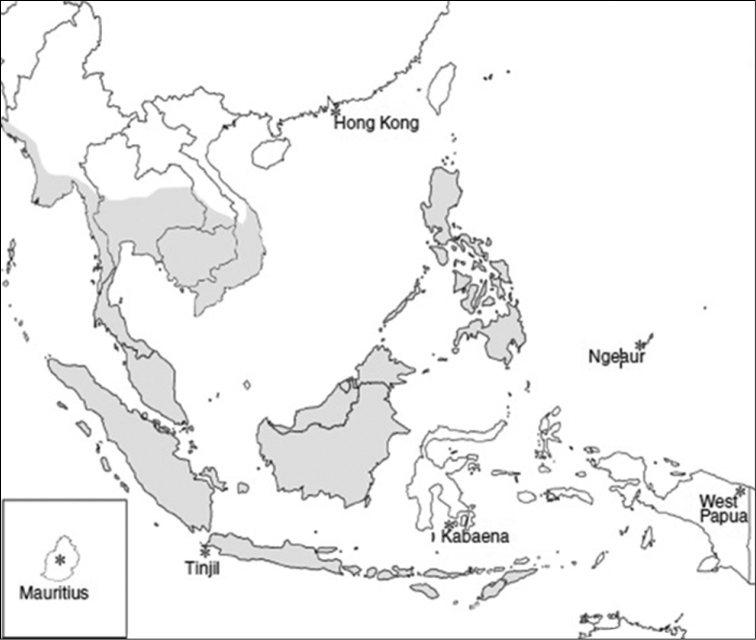
Distribution of the long-tailed macaque (*Macaca fascicularis*) in Southeast Asia (Gumert et al. 2011).

*Macaca fascicularis* (Raffles, 1821) is also known as long-tailed, crab-eating or cynomolgus macaque. This species is well distributed in the countries of Malaysia, Brunei, Bangladesh, Cambodia, Nicobar Islands, Indonesia, Lao PDR, Myanmar, Philippines, Singapore, Thailand, Timor-Leste and Vietnam (Figure 1) (Gumert et al. 2011). There appears to be a hybrid zone between *Macaca fascicularis* and *Macaca mulatta* (Zimmermann, 1780) in the northern range above mainland Southeast Asia, which makes it difficult to determine the northern distribution limit of *Macaca fascicularis* (Fooden 1996). The distribution of long-tailed macaques was extended to the Pacific Ocean (Palau) (Crombie and Pregill 1999), Indian Ocean (Mauritius) (Trask et al. 2013) and New Guinea (Kemp and Burnett 2003) due to human-mediated introduction of the species to these respective regions in the recent past.

At least 10 subspecies of *Macaca fascicularis* are presently recognized; *Macaca fascicularis atriceps* (Kloss, 1919), *Macaca fascicularis aurea* (Geoffroy, 1831), *Macaca fascicularis condorensis* (Kloss, 1926), *Macaca fascicularis fascicularis* (Raffles, 1821), *Macaca fascicularis fusca* (Miller, 1903), *Macaca fascicularis karimondjawae* (Sody, 1949), *Macaca fascicularis lasiae* (Lyon, 1916), *Macaca fascicularis philippinensis* (Geoffroy, 1843), *Macaca fascicularis tua* (Kellog, 1944) and *Macaca fascicularis umbrosa* (Miller, 1902) (Groves 2001; Brandon-Jones et al. 2004) based on their morphological characteristics. These subspecies classifications were distinguished based on three critical aspects: tail length, pelage coloration and form of the cheek whiskers (Groves 2001). Both Groves (2001) and Brandon-Jones et al. (2004) agreed that only one subspecies, *Macaca fascicularis fascicularis*, is distributed in Peninsula Malaysia and Borneo. Medway (1969), on the other hand, has acknowledged 3 subspecies of *Macaca fascicularis* distributed in Malaysia, specifically, *Macaca fascicularis fascicularis* (Raffles, 1821) (Peninsula Malaysia); *Macaca fascicularis argentimembris* (Kloss, 1911) (Redang Island) and *Macaca fascicularis laeta* (Elliot, 1909) (Tioman Island and Tinggi Island). Raven (1935) acknowledged *Macaca fascicularis argentimembris* by Kloss, 1911 observed in Redang Island as subspecies distributed in East Coast of Peninsula Malaysia. Weitzel et al. (1988) also acknowledged the distribution of *Macaca fascicularis laeta* by Elliot (1909) observed in Tioman Island and Tinggi Island as subspecies distributed in the East Coast of Peninsula Malaysia.

Zhang et al. (1993) conducted one of the earliest thorough studies on the phylogeny of *Macaca fascicularis* that exploited mitochondrial DNA (mtDNA) using restriction endonuclease analysis. Smith et al. (2007) studied mtDNA variation within and among regional populations of *Macaca fascicularis* by applying an astonishing 1053 samples comprising 5 regional populations (Malaysia, Indonesia, Indochina, the Philippines and Mauritius). Deinard and Smith (2001) screened the nuclear DNA sequences (natural resistance-associated macrophage protein 1, *NRAMP1*) of 59 individuals representing 11 species of macaques, and their results suggest that *Macaca fascicularis* may not be as primitive as the mtDNA data suggests. Numerous other genetic studies on *Macaca fascicularis* have been conducted. Tosi et al. (2002) determined the introgression between *Macaca fascicularis* and *Macaca mulatta* using Y-chromosome and mitochondrial markers. Otting et al. (2009) studied the haplotypes in pedigreed cynomolgus macaques. Street et al. (2007) analyzed the nucleotide polymorphisms in *Macaca mulatta* and *Macaca fascicularis*. Blancher et al. (2008) described the phylogeny of 4 populations of *Macaca fascicularis fascicularis* (Indonesia, Indochina, Philippines and Mauritius). Stevison and Kohn (2009) conducted genetic analysis to determine hybridization between rhesus and long-tailed macaques. Finally, Md-Zain et al. (2010a) determined the phylogenetic relationships of Cercopithecidae using *Macaca fascicularis* as a representative.

Despite the abundance of genetic studies on or including *Macaca fascicularis*, the phylogenetic relationships among Malaysia’s long-tailed macaques remain uncertain. However, these phylogenetic data are crucial for conservation management of *Macaca fascicularis* as this species is reported as a pest in human settlement areas (Md-Zain et al. 2010b; 2011). For example, *Macaca fascicularis* engages frequently in crop-raiding activities, and these behaviors are often reinforced by humans that feed these macaques either directly or indirectly, which leads to unintentional habituation of the species. The annual report by the Department of Wildlife and National Parks (PERHILITAN) (2010) indicated that *Macaca fascicularis* is at the top of the human-wildlife conflict species case list. From a recorded 9,286 complaints of wildlife disturbance from various species, complaints on *Macaca fascicularis* disturbance were the highest, with 5,930 complaints (63.86%). The phylogenetic relationships of Malaysia’s *Macaca fascicularis* data are crucial in planning and executing the translocation process of this species in the future, which is one of the major actions in the conservation management and human-wildlife conflict management of the species. By understanding the phylogenetic relationships of Malaysia’s crab-eating macaque, the plan for translocation the species can finally be carried out without a risk of losing important genetic diversity or even unique genetic lineages of the species.

Phylogenetic studies at the subspecies level are very scarce (Rosli et al. 2014), as many primatologists are making the species group classifications the focal point in their studies. Both Vun et al. (2011) and Blancher et al. (2008) have successfully explained the phylogenetic relationships of *Presbytis* and *Macaca fascicularis*, respectively, at population level using Cyt *b* sequences, proving that Cyt *b* is a suitable locus for population level studies of Cercopithecidae. Thus, the objectives of this research are to determine the phylogenetic relationships of Malaysia’s *Macaca fascicularis* using mitochondrial Cyt *b* sequences and to test genetically the subspecies classifications as made by Medway (1969), Groves (2001) and Brandon-Jones et al. (2004) based on morphological characteristics.

## Methods

### DNA extraction, polymerase chain reaction (PCR) and sequencing

**Figure 2.**
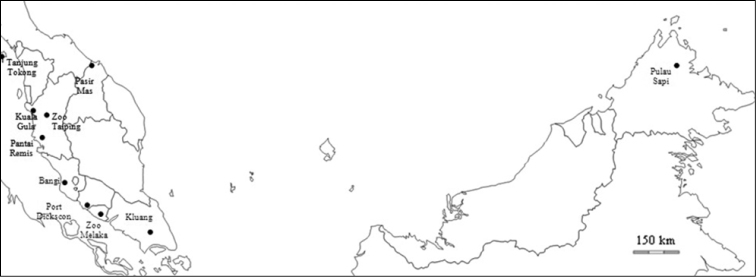
Sampling location of *Macaca fascicularis* throughout Peninsula Malaysia and Borneo.

Altogether, 27 genetic samples (Table 1 and Figure 2) were used in this research. These samples were provided by the Department of Wildlife and National Parks (PERHILITAN) and Sabah Parks. The samples derive mainly from feces collected in the original habitats of *Macaca fascicularis*. In addition, blood and tissue samples collected from a roadkill specimen of *Macaca fascicularis* were also used in this study. Mitochondrial DNA (mtDNA) was extracted from each genetic sample using QIAGEN DNeasy Blood and Tissue Kit, following the manufacturer’s protocol. A mtDNA genome from FTA (fast technology for analysis of nucleic acids) sample was extracted using the WHATMAN^®^ GenSolve Recovery Kit, also following the manufacturer’s protocol. DNA was extracted from 0.5 g – 1.0 g of fecal sample using innuPREP Stool DNA kit (Analytik Jena) following the manufacturer’s protocol.

**Table 1.** Details on the samples used in this study.

No.	Sample name	Taxon	Locality
1	MF135	*Macaca fascicularis fascicularis*	Port Dickson, Negeri Sembilan
2	MF136	*Macaca fascicularis fascicularis*	Port Dickson, Negeri Sembilan
3	MF137	*Macaca fascicularis fascicularis*	Port Dickson, Negeri Sembilan
4	MF138	*Macaca fascicularis fascicularis*	Port Dickson, Negeri Sembilan
5	M1	*Macaca fascicularis fascicularis*	Bangi, Selangor
6	BM95	*Macaca fascicularis fascicularis*	Kluang, Johor
7	MF488	*Macaca fascicularis fascicularis*	Tanjung Tokong. Pulau Pinang
8	MF489	*Macaca fascicularis fascicularis*	Tanjung Tokong. Pulau Pinang
9	MF490	*Macaca fascicularis fascicularis*	Tanjung Tokong. Pulau Pinang
10	MF491	*Macaca fascicularis fascicularis*	Tanjung Tokong. Pulau Pinang
11	MF719	*Macaca fascicularis fascicularis*	Pantai Remis, Perak
12	MF720	*Macaca fascicularis fascicularis*	Pantai Remis, Perak
13	MF721	*Macaca fascicularis fascicularis*	Pantai Remis, Perak
14	MF722	*Macaca fascicularis fascicularis*	Pantai Remis, Perak
15	ALMFD16	*Macaca fascicularis fascicularis*	Pasir Mas, Kelantan
16	ALMFD17	*Macaca fascicularis fascicularis*	Pasir Mas, Kelantan
17	ALMFD28	*Macaca fascicularis fascicularis*	Pasir Mas, Kelantan
18	ALMFD29	*Macaca fascicularis fascicularis*	Pasir Mas, Kelantan
19	ALMFA62	*Macaca fascicularis fascicularis*	Kuala Gula, Perak
20	ALMFA63	*Macaca fascicularis fascicularis*	Kuala Gula, Perak
21	ALMFA64	*Macaca fascicularis fascicularis*	Kuala Gula, Perak
22	ALMFA65	*Macaca fascicularis fascicularis*	Kuala Gula, Perak
23	MF03	*Macaca fascicularis fascicularis*	Pulau Sapi, Sabah
24	MF04	*Macaca fascicularis fascicularis*	Pulau Sapi, Sabah
25	MF05	*Macaca fascicularis fascicularis*	Pulau Sapi, Sabah
26	BM97	*Macaca nemestrina*	Zoo Taiping, Perak
27	BM104	*Macaca arctoides*	Malacca Zoo

Polymerase Chain Reaction (PCR) was employed in order to amplify the targeted locus in the mtDNA genome, which is a partial fragment of the Cyt *b* gene, by using Mastercycler® nexus (Eppendorf North America, Inc.). PCR was performed by using Phusion^TM^ Flash High-Fidelity PCR Master Mix (Finnzymes, OY), which has extreme speed (extension times of 15 s/kb or less), high accuracy (proofreads DNA polymerase with a fidelity of 25 X *Taq polymerase*) and a very high yield in reduced times. Primers used in this study were (L14724) 5’- CGAAGCTTGATATGAAAAACCATCGTTG -3’ (Pääbo and Wilson 1988) and (H15149) 5’- AAACTGCAGCCCCTCAGAATGATATTTGTCCTCA - 3’ (Kocher et al. 1989). PCR reactions were carried out under the following parameters: 98°C initial denaturation for 30 seconds, followed by 30 cycles of 98°C denaturation for 10 seconds, 55°C of annealing for 30 seconds, 72°C extension for 30 seconds and the final extension stage at 72°C for 10 minutes. The PCR product was purified using the Vivantis G-F1 PCR Clean-up Kit, and the purified PCR products were sent to the 1^st^ Base Laboratories Sdn Bhd (Malaysia) for sequencing.

### Sequence and phylogenetic analysis

Sequence results obtained from the 1^st^ Base Laboratories Sdn Bhd were proofread and edited using Bioedit Sequence Alignment Editor, and the Sequence Similarity searches were performed using GenBank BLASTn application to validate the DNA sequences obtained. DNA sequences were submitted to GenBank under accession number KJ592589-KJ592594. Bioedit’s ClustalW multiple alignment algorithm was then used to align the sequence results, and sequence analysis and phylogenetic analysis were performed. DNASP 4.0 (Rozas et al. 2003), PAUP 4.0b10 (Swofford 2002) and MEGA version 4.0 (Tamura et al. 2007) software were used for sequence analysis to determine nucleotide diversity (π) and net nucleotide divergence (Da); genetic distance and single nucleotide polymorphisms (SNPs) of the sequences/datasets respectively.

Three methods of phylogenetic tree reconstructions were carried out; the distance-based method (*neighbor joining*, NJ) using MEGA version 4.0 (Tamura et al. 2007); the character-based method (*maximum parsimony*, MP) using Phylogenetic Analysis Using Parsimony (PAUP) version 4.0b10 (Swofford 2002) and Bayesian inference using MrBayes 3.1 (Huelsenbeck and Ronquist 2001). The Kimura-2-Parameter model was selected for NJ phylogenetic reconstructions. MP phylogenetic tree was carried out with heuristic search methods and 1000 random stepwise addition with the application of a 50% consensus-majority rule concept (Swofford 2002). In the MP analysis, each transition and transversion was calculated on average. The MP phylogenetic tree was constructed using a tree bisection and reconnection (TBR) algorithm, and all the trees constructed underwent 1000 bootstrap replications to obtain the bootstrap confidence level.

Modeltest version 3.7 software (Posada and Crandall 1998) was used to select the best substitution model for the partial Cyt *b* sequences using Akaike Information Criterion (AIC). The best substitution model was applied in the Bayesian analysis using MrBayes 3.1.2. software. The most suitable model that fit the data was the HKY+G model with a gamma shape parameter of 0.5455 and base frequencies of 0.2866 for A, 0.3171 for C, 0.1266 for G and 0.2696 for T. We ran Metropolis-coupled Markov Chain Monte Carlo (MCMC) with 300000 generations, with 0.008214 Split Frequencies Probability (P) and tree was sampled every 10 generations. The first 25% of the trees obtained in the analysis was discarded as burn-in (7500 trees discarded from total 30000 trees), a majority-rule consensus of remaining trees was constructed and posterior probabilities (PP) were summarized for each branch.

## Results

### Comparison of the cytochrome *b* sequence of Malaysia’s *Macaca fascicularis* with GenBank’s sequence

Partial sequences of Cyt *b* locus in size of 383 bp were successfully sequenced for all 27 genetic samples (Table 1). The first analysis conducted on the sequences consisted of sequence similarity searches using the GenBank application to validate each sequence obtained was from the correct taxon of the acquired samples and to avoid encountering the data problem of nuclear insertion. All genetic samples matched the target species sequences in GenBank with samples FJ906803.1 (*Macaca fascicularis* complete genome) and corresponded to most of the ingroup samples with average query cover and maximum identities scores at 95% and 97%, respectively.

### Sequence polymorphism, genetic distance and nucleotide diversity

A total of 27 genetic sequences in a size of 383 bp of Cyt *b* locus yielded 78 (20.37%) variable sites, of which 34 sites were parsimony informative characters (8.88%). Interestingly, when outgroup samples (*Macaca nemestrina* and *Macaca arctoides*) were excluded from the analysis, only 23 (6%) variable sites were detected, all of which were parsimony informative characters. From these 23 informative characters, 13 were generated by the inclusion of Borneo samples in the analysis; whereas, if the Borneo samples were excluded, only 10 (2.6%) parsimony informative characters were detected.

Pairwise genetic distances of Cyt *b* partial sequences were calculated with PAUP 4.0b10 (Swofford 2002) using the Kimura-2-Parameter model (Table 2). The genetic distance of samples originating from Borneo and Peninsula Malaysia showed a minimum genetic distance of 0.04068 (BM95) and a maximum value as high as 0.049 (ALMFA62, ALMFA63, ALMFA64 and ALMFA65), which is the highest value within the *Macaca fascicularis* genetic distance analysis. The genetic distance of samples originating from the East Coast of Peninsula Malaysia and West Coast of Peninsula Malaysia (excluding samples from Borneo) showed the minimum value of genetic distance was 0.008 (BM95), and the maximum value was 0.016 (ALMFA62, ALMFA63, ALMFA64 and ALMFA65). The separation of the island population of *Macaca fascicularis* and the mainland of Peninsula Malaysia populations (excluding samples from Borneo) showed a minimum value of genetic distance of 0.008 (MF719, MF720, MF721 and MF722) and a maximum value of genetic distance of 0.016 (MF135, MF136, MF137 and MF138 and M1).

**Table 2.** Pairwise distance of *Macaca fascicularis* samples based on Kimura-2-Parameter algorithm model.

		1	2	3	4	5	6	7	8	9	10	11	12	13	14
**1**	MF03 Mf Sabah	-													
**2**	MF04 Mf sabah	0.00000	-												
**3**	MF05 Mf Sabah	0.00000	0.00000	-											
**4**	BM95 Mf Johor	0.04068	0.04068	0.04068	-										
**5**	MF135 Mf NSembil	0.04636	0.04636	0.04636	0.00525	-									
**6**	MF136 Mf Nsembil	0.04636	0.04636	0.04636	0.00525	0.00000	-								
**7**	MF137 Mf Nsembil	0.04636	0.04636	0.04636	0.00525	0.00000	0.00000	-							
**8**	MF138 Mf Nsembi	0.04636	0.04636	0.04636	0.00525	0.00000	0.00000	0.00000	-						
**9**	M1 Mf Selangor	0.04636	0.04636	0.04636	0.00525	0.00000	0.00000	0.00000	0.00000	-					
**10**	MF719 Mf Perak	0.04351	0.04351	0.04351	0.00262	0.00789	0.00789	0.00789	0.00789	0.00789	-				
**11**	MF720 Mf Perak	0.04351	0.04351	0.04351	0.00262	0.00789	0.00789	0.00789	0.00789	0.00789	0.00000	-			
**12**	MF721 Mf Perak	0.04351	0.04351	0.04351	0.00262	0.00789	0.00789	0.00789	0.00789	0.00789	0.00000	0.00000	-		
**13**	MF722 Mf Perak	0.04351	0.04351	0.04351	0.00262	0.00789	0.00789	0.00789	0.00789	0.00789	0.00000	0.00000	0.00000	-	
**14**	ALMFA65 Mf Perak	0.04923	0.04923	0.04923	0.00789	0.01323	0.01323	0.01323	0.01323	0.01323	0.00525	0.00525	0.00525	0.00525	-
**15**	ALMFA64 Mf Perak	0.04923	0.04923	0.04923	0.00789	0.01323	0.01323	0.01323	0.01323	0.01323	0.00525	0.00525	0.00525	0.00525	0.00000
**16**	ALMFA63 Mf Perak	0.04923	0.04923	0.04923	0.00789	0.01323	0.01323	0.01323	0.01323	0.01323	0.00525	0.00525	0.00525	0.00525	0.00000
**17**	ALMFA62 Mf Perak	0.04923	0.04923	0.04923	0.00789	0.01323	0.01323	0.01323	0.01323	0.01323	0.00525	0.00525	0.00525	0.00525	0.00000
**18**	MF488 Mf P.Pinan	0.04636	0.04636	0.04636	0.01055	0.01592	0.01592	0.01592	0.01592	0.01592	0.00789	0.00789	0.00789	0.00789	0.01323
**19**	MF489 Mf P.Pinan	0.04636	0.04636	0.04636	0.01055	0.01592	0.01592	0.01592	0.01592	0.01592	0.00789	0.00789	0.00789	0.00789	0.01323
**20**	MF490 Mf P.Pinan	0.04636	0.04636	0.04636	0.01055	0.01592	0.01592	0.01592	0.01592	0.01592	0.00789	0.00789	0.00789	0.00789	0.01323
**21**	MF491 Mf P.Pinan	0.04636	0.04636	0.04636	0.01055	0.01592	0.01592	0.01592	0.01592	0.01592	0.00789	0.00789	0.00789	0.00789	0.01323
**22**	ALMFD16 Mf Kelan	0.03786	0.03786	0.03786	0.00789	0.01323	0.01323	0.01323	0.01323	0.01323	0.01055	0.01055	0.01055	0.01055	0.01592
**23**	ALMFD17 Mf Kelan	0.03786	0.03786	0.03786	0.00789	0.01323	0.01323	0.01323	0.01323	0.01323	0.01055	0.01055	0.01055	0.01055	0.01592
**24**	ALMFD28 Mf Kelan	0.03786	0.03786	0.03786	0.00789	0.01323	0.01323	0.01323	0.01323	0.01323	0.01055	0.01055	0.01055	0.01055	0.01592
**25**	ALMFD29 Mf Kelan	0.03786	0.03786	0.03786	0.00789	0.01323	0.01323	0.01323	0.01323	0.01323	0.01055	0.01055	0.01055	0.01055	0.01592
**26**	BM104 Ma Malacca	0.11558	0.11558	0.11558	0.11914	0.12574	0.12574	0.12574	0.12574	0.12574	0.12243	0.12243	0.12243	0.12243	0.12907
**27**	BM97 Mn ZooTaipi	0.11753	0.11753	0.11753	0.13751	0.14426	0.14426	0.14426	0.14426	0.14426	0.14087	0.14087	0.14087	0.14087	0.14087

**Table 2.** Continue.

		15	16	17	18	19	20	21	22	23	24	25	26	27
**1**	MF03 Mf Sabah													
**2**	MF04 Mf sabah													
**3**	MF05 Mf Sabah													
**4**	BM95 Mf Johor													
**5**	MF135 Mf NSembil													
**6**	MF136 Mf Nsembil													
**7**	MF137 Mf Nsembil													
**8**	MF138 Mf Nsembi													
**9**	M1 Mf Selangor													
**10**	MF719 Mf Perak													
**11**	MF720 Mf Perak													
**12**	MF721 Mf Perak													
**13**	MF722 Mf Perak													
**14**	ALMFA65 Mf Perak													
**15**	ALMFA64 Mf Perak	-												
**16**	ALMFA63 Mf Perak	0.00000	-											
**17**	ALMFA62 Mf Perak	0.00000	0.00000	-										
**18**	MF488 Mf P.Pinan	0.01323	0.01323	0.01323	-									
**19**	MF489 Mf P.Pinan	0.01323	0.01323	0.01323	0.00000	-								
**20**	MF490 Mf P.Pinan	0.01323	0.01323	0.01323	0.00000	0.00000	-							
**21**	MF491 Mf P.Pinan	0.01323	0.01323	0.01323	0.00000	0.00000	0.00000	-						
**22**	ALMFD16 Mf Kelan	0.01592	0.01592	0.01592	0.01323	0.01323	0.01323	0.01323	-					
**23**	ALMFD17 Mf Kelan	0.01592	0.01592	0.01592	0.01323	0.01323	0.01323	0.01323	0.00000	-				
**24**	ALMFD28 Mf Kelan	0.01592	0.01592	0.01592	0.01323	0.01323	0.01323	0.01323	0.00000	0.00000	-			
**25**	ALMFD29 Mf Kelan	0.01592	0.01592	0.01592	0.01323	0.01323	0.01323	0.01323	0.00000	0.00000	0.00000	-		
**26**	BM104 Ma Malacca	0.12907	0.12907	0.12907	0.12574	0.12574	0.12574	0.12574	0.10940	0.10940	0.10940	0.10940	-	
**27**	BM97 Mn ZooTaipi	0.14087	0.14087	0.14087	0.13751	0.13751	0.13751	0.13751	0.13416	0.13416	0.13416	0.13416	0.14702	-

Nucleotide diversity (π) and net nucleotide divergence (Da) were also calculated for the Cyt *b* sequence obtained using DnaSP v4.0. Two separate analyses for π and Da were conducted based on the origin of samples, by first sorting the sequences (excluding outgroup) according to their locality (states) (Table 3) and then according to regions (Table 4). The first analysis (Table 3) portrayed that π was the highest between Sabah and other states ranging from 0.019 to 0.025, and the results are consistent with Da, ranging from 0.037 to 0.044. π. The lowest values, 0 for π and Da, were found between Negeri Sembilan and Selangor. The second analysis (Table 4) revealed that the Peninsula Malaysia and Borneo populations have 0.017 π and 0.038 Da, which are the highest compared to other regions; the East Coast of Peninsula Malaysia and West Coast of Peninsula Malaysia populations have 0.09 π and 0.08 Da and mainland and island populations have 0.08 π and 0.09 Da, respectively.

**Table 3.** Measures of nucleotide diversity (π) and net nucleotide divergence among populations of *Macaca fascicularis* analyzed by locality.

Locality	Nucleotide Diversity (π)	Net nucleotide divergence (Da)
Sabah-Johor	0.01958	0.03916
Sabah-Negeri Sembilan	0.02536	0.04439
Sabah-Selangor	0.02219	0.04439
Sabah-Perak	0.02089	0.04289
Sabah-Pulau Pinang	0.02536	0.04439
Sabah-Kelantan	0.02089	0.03655
Johor-Negeri Sembilan	0.00209	0.00522
Johor-Selangor	0.00522	0.00522
Johor-Perak	0.00348	0.00373
Johor-Pulau Pinang	0.00418	0.01044
Johor-Kelantan	0.00313	0.00783
Negeri Sembilan-Selangor	0.00000	0.00000
Negeri Sembilan-Perak	0.00633	0.00895
Negeri Sembilan-Pulau Pinang	0.00895	0.01567
Negeri Sembilan-Kelantan	0.00746	0.01305
Selangor-Perak	0.00464	0.00895
Selangor-Pulau Pinang	0.00627	0.01567
Selangor-Kelantan	0.00522	0.01305
Perak-Pulau Pinang	0.00633	0.00895
Perak-Kelantan	0.00760	0.01156
Pulau Pinang-Kelantan	0.00746	0.01305

**Table 4.** Measures of nucleotide diversity (π) and net nucleotide divergence among populations of *Macaca fascicularis* analyzed by regions.

Regions	Nucleotide Diversity (π)	Net nucleotide divergence (Da)
Peninsula Malaysia-Borneo	0.01666	0.03801
East Coast-West Coast	0.00943	0.00865
Mainland-Island	0.00823	0.00918

## Phylogenetic trees

### Neighbor joining

**Figure 3.**
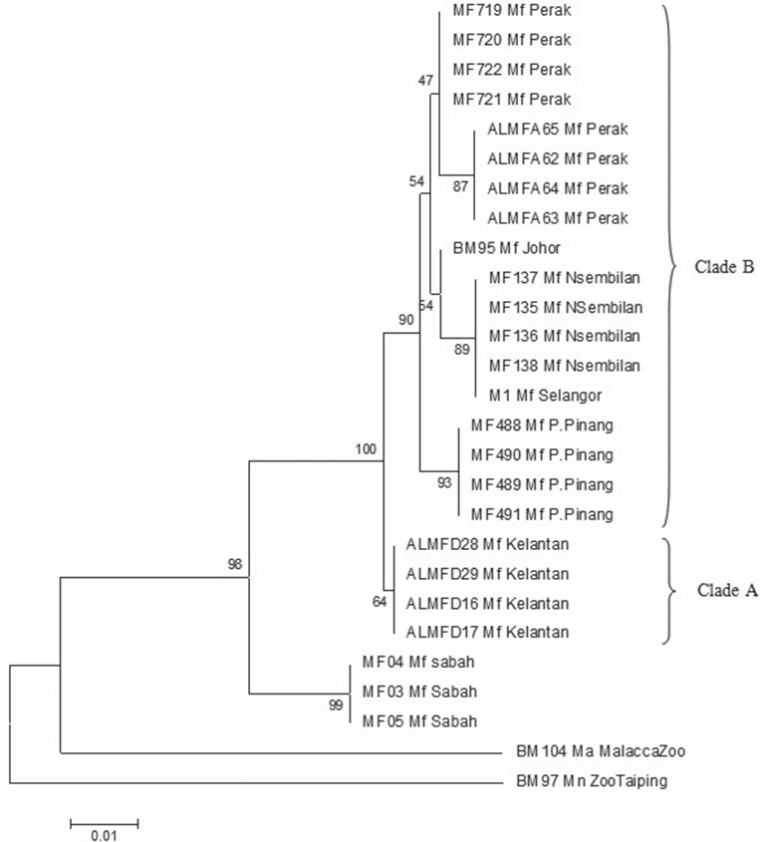
*Neighbor joining* phylogenetic tree using Kimura-2-Parameter algorithm with bootstrap values indicated on the branch.

The NJ phylogeny tree (Figure 3) was generated using Kimura-2-Parameter with 1000 bootstrap replication. The NJ phylogenetic tree showed that samples originating from Borneo remain monophyletic from samples originating from Peninsula Malaysia, supported with 99% bootstrap value. Samples from Peninsula Malaysia were divided into 2 clades; clade A and Clade B. Clade A portrays the separation of samples originating from the East Coast of Peninsula Malaysia from the remaining samples with 64% bootstrap value. Clade B on the other hand is the assemblage of populations of the West Coast of Peninsula Malaysia, supported by 90% bootstrap value. Within Clade B, two further clades were defined, namely island population (Pulau Pinang) and mainland populations (Perak, Negeri Sembilan, Johor, and Selangor) supported by 93% and 54% bootstrap value respectively. Populations from southern Peninsula (Negeri Sembilan, Johor and Selangor) and northern Peninsula (Perak) also were distinguished by 54% and 47% bootstrap value correspondingly.

### Maximum parsimony

**Figure 4.**
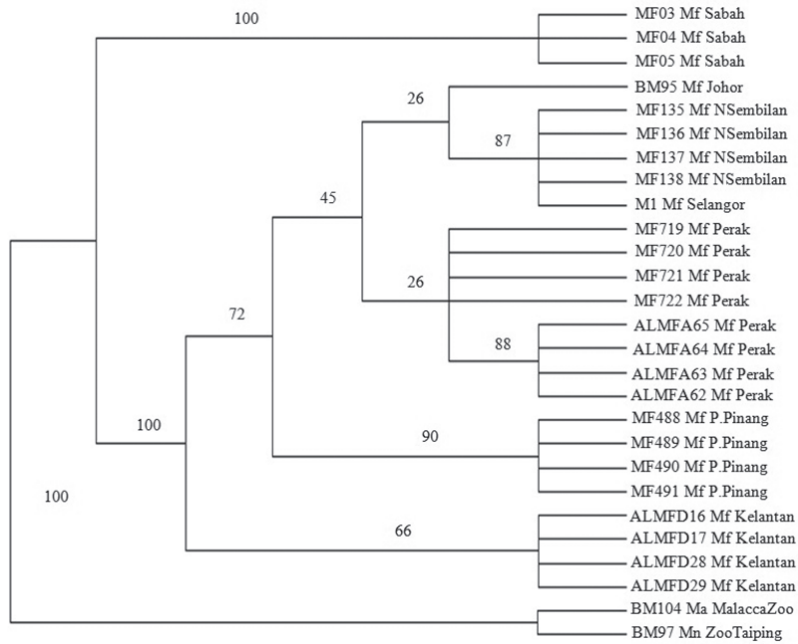
The Bootstrap 50% majority rule consensus maximum parsimony tree of *Macaca fascicularis* populations. Bootstrap values are indicated on the branch.

MP (Figure 4) analysis was conducted using PAUP 4.0 (CI = 0.929, HI = 0.071, RI = 0.944, RC = 0.878 and tree length = 85). *Macaca fascicularis* populations were separated into 2 main clades, a Borneo clade and a Peninsula Malaysia clade both supported by 100% bootstrap value in the MP tree. Peninsula Malaysia’s population was further divided into 2 sub clades, the East Coast of Peninsula Malaysia and West Coast of Peninsula Malaysia populations supported by 66% and 72% bootstrap value respectively. The West Coast of Peninsula Malaysia populations were further divided into 2 clades, comprising the mainland and island populations (Pulau Pinang) supported by 45% and 90% bootstrap value. The Northern Peninsula (Perak) and Southern Peninsula (Selangor, Negeri Sembilan and Johor) clades were also separated, both supported by 26% bootstrap value.

### Bayesian inference

**Figure 5.**
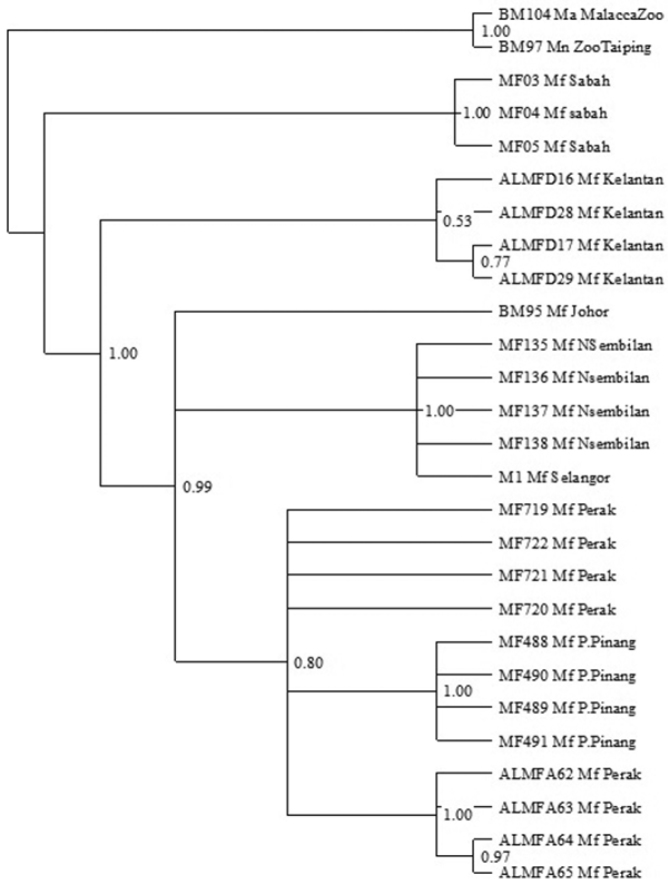
Bayesian inference of the 50% majority rule consensus tree of Cyt *b* sequence of *Macaca fascicularis* populations with Bayesian posterior probability (PP) are accordingly indicated on the branch.

Bayesian inference phylogenetic tree (Figure 5) results are generally consistent with neighbor joining and maximum parsimony phylogenetic trees. Separations of *Macaca fascicularis* populations originating from Borneo and Peninsula Malaysia correspond well with other phylogenetic trees. Within *Macaca fascicularis* populations from Peninsula Malaysia, the East Coast of Peninsula Malaysia populations are yet again separated from the West Coast of Peninsula Malaysia populations with 0.53 and 0.99 posterior probabilities respectively. The island populations (Pulau Pinang), on the other hand, seem to group together with other mainland populations from the West Coast of Peninsula Malaysia. These are thoroughly inconsistent with both neighbor joining and maximum parsimony phylogenetic trees. The populations from Negeri Sembilan, Selangor and Johor are also distinguished from *Macaca fascicularis* populations from Perak and Pulau Pinang, supported by a 1.00 and 0.80 posterior probability.

## Discussion

Sequence analysis of partial Cyt *b* sequences indicates that this locus is highly conserved which yielded only 78 (20.37%) variable sites among which only 34 (8.88%) of parsimony informative characters across *Macaca fascicularis*, *Macaca nemestrina* and *Macaca arctoides*. Vun et al. (2011) reported 156 (40.21%) variable sites and 124 (31.96%) parsimony informative sites in the partial Cyt *b* gene (388 bp) across *Presbytis* and *Trachypithecus* (Colobinae). These findings are much higher compared to findings in this research, although analysis in this research was done within one genus while Vun et al. (2011) analyzed across 2 genera. Haus et al. (2013) analyzed 28.86% variable sites and 19.82% parsimony informative sites from full Cyt *b* sequences in *Chlorocebus*, which are parallel with our findings, indicating Cyt *b* is a highly conserved gene. Mitochondria are well known to play a central role in a variety of cellular processes and are the main source of adenosine triphosphate (ATP) (Kujoth et al. 2005). Cyt *b* is a functional gene that contains both redox centers, Q_0_ and Q_i_, involved in electron transfer (Hatefi 1985; Howell and Gilbert 1988). Thus, even a single point mutation that occurs in this region will probably have a deleterious effect on the functional response of the locus.

*Macaca fascicularis* populations in Malaysia are evidently divided into 2 main clusters: the Borneo populations and Peninsula Malaysia populations. Thirteen parsimony informative characters were detected between these populations from a total of 23 parsimony informative characters. Pairwise genetic distance analysis indicated that the genetic distance between these 2 populations was the highest as compared to other populations in Malaysia with a value of 0.041-0.049, aside from monophyletic states of both populations in all phylogenetic trees. These findings are highly anticipated, considering the vicariance theory on population disjunction as the central thesis. In this scenario, the Borneo populations and Peninsula Malaysia populations are separated by the South China Sea. This separation more than likely caused the interruption of gene flow between both populations, causing them to accrue the major genetic differences observed in this study.

Populations of *Macaca fascicularis* in Peninsula Malaysia also form 2 further subgroups: east and west. The population from Kelantan forms a single clade in all phylogenetic trees and genetic analysis in comparison to the rest of the Peninsula Malaysia populations. Thus, the Kelantan population likely represents a unique lineage as compared to other populations from Peninsula Malaysia. Results obtained by Vun et al. (2011) that used the same primers as in this research to study the phylogenetic relationships of genus *Presbytis* in Malaysia provide a suitable comparison. Vun et al. (2011) obtained a pairwise genetic distance (Kimura-2-Parameter) between *Presbytis melalophos robinsoni* and *Presbytis melalophos siamensis* as low as 0.058, and each of these subspecies has uniquely distinct morphological characteristics. In comparison, the pairwise genetic distance observed here between *Macaca fascicularis* populations of East and West coast of Peninsula Malaysia are 0.016; however, there are no documented morphological characteristics that differentiate these populations.

The observed genetic differences between populations of *Macaca fascicularis* are best viewed as subspecies separations. *Macaca fascicularis laeta* (Elliot, 1909) was recorded by Medway (1969) as common on Tioman Island (the East Coast of Peninsula Malaysia), but a survey conducted by the Department of Wildlife and Nature Park, Malaysia, PERHILITAN (1995) proved otherwise. This particular subspecies was observed based on morphological characters on the mainland East Coast of Peninsula Malaysia by PERHILITAN (1995) as well as Weitzel et al. (1988), whose study also acknowledged the distribution of *Macaca fascicularis laeta* in the East Coast of Peninsula Malaysia. Raven (1935), however, acknowledges *Macaca fascicularis argentimembris* (Kloss, 1911) observed in Redang Island as the subspecies distributed in the East Coast of Peninsula Malaysia. Although these 2 classifications contradict each other on one dimension, both may be true. It may be possible that *Macaca fascicularis argentimembris* are distributed in the North East Coast of Peninsula Malaysia and *Macaca fascicularis laeta* are distributed in the South East Coast of Peninsula Malaysia. These are the most relevant classifications according to phylogenetic and genetic results of this study, which suggests that the Pasir Mas’ population is *Macaca fascicularis argentimembris*. In contrast, the populations of Kluang, Pantai Remis, Kuala Gula, Port Dickson, and Bangi distributed on the west coast should be *Macaca fascicularis fascicularis*.

The results of this study are directly relevant to conservation management strategies of *Macaca fascicularis* in Malaysia, particularly in terms of the translocation process. The human-macaque conflict has intensified in Malaysia due to habitat degradation and habituation of the species. It is important to recognize the genetic diversity between populations of *Macaca fascicularis* so we are able to conserve the unique evolutionary lineages of the species. Thus, PERHILITAN can translocate the target populations of *Macaca fascicularis* within the same gene pool of the other populations (the Borneo-Peninsula; east-west; mainland-island and north-south populations). This translocation is important to avoid the loss of genetic diversity that cannot be recovered, or, in extreme cases, can result in hybridization of populations and potential outbreeding depression of the populations (DeSalle and Amato 2004).

## Conclusion

The phylogenetic relationships of Malaysia’s long-tailed macaques, *Macaca fascicularis*, are crucial, as they are at a center of the human-wildlife crisis in the field of primatology, taxonomy and conservation biology. This research has shown that populations of *Macaca fascicularis* originating from Borneo and Peninsula Malaysia are distinguishable from each other based on genetic data. Within Peninsula Malaysia, further division occurs between the East Coast and West Coast of Peninsula Malaysia; mainland and island and Northern and Southern Peninsula populations. These data can aid the conservation management planning in terms of translocation of the target pest populations within similar gene pools. Further mtDNA and nuclear DNA studies of *Macaca fascicularis* at the population level are required to increase the number of individuals from each locality and increase the number of geographical locations to represent clades (east-west and Peninsula-Borneo), in which biogeographical factors can also be taken into account.
